# Comparative Analysis of the Interaction between Different Flavonoids and PDIA3

**DOI:** 10.1155/2016/4518281

**Published:** 2016-12-04

**Authors:** Flavia Giamogante, Ilaria Marrocco, Donatella Romaniello, Margherita Eufemi, Silvia Chichiarelli, Fabio Altieri

**Affiliations:** ^1^Department of Biochemical Sciences “A. Rossi Fanelli”, Sapienza University, P.le A. Moro 5, 00185 Rome, Italy; ^2^Istituto Pasteur-Fondazione Cenci Bolognetti, Sapienza University, P.le A. Moro 5, 00185 Rome, Italy

## Abstract

Flavonoids, plant secondary metabolites present in fruits, vegetables, and products such as tea and red wine, show antioxidant, anti-inflammatory, antithrombotic, antiviral, and antitumor activity. PDIA3 is a member of the protein disulfide isomerase family mainly involved in the correct folding of newly synthetized glycoproteins. PDIA3 is associated with different human pathologies such as cancer, prion disorders, Alzheimer's disease, and Parkinson's diseases and it has the potential to be a pharmacological target. The interaction of different flavonoids with PDIA3 was investigated by quenching fluorescence analysis and the effects on protein activity were evaluated. A higher affinity was observed for eupatorin-5-methyl ether and eupatorin which also inhibit reductase activity of PDIA3 but do not significantly affect its DNA binding activity. The use of several flavonoids differing in chemical structure and functional groups allows us to make some consideration about the relationship between ligand structure and the affinity for PDIA3. The specific flavone backbone conformation and the degree of polarity seem to play an important role for the interaction with PDIA3. The binding site is probably similar but not equivalent to that of green tea catechins, which, as previously demonstrated, can bind to PDIA3 and prevent its interaction with DNA.

## 1. Introduction

PDIA3/ERp57 is a member of the protein disulfide isomerase family mainly present in the endoplasmic reticulum, where it carries out a major role as a key molecular player in the quality control and correct folding of newly synthesized glycoproteins [1]. However, PDIA3 has been found in different subcellular compartments where it has been proposed and sometimes proved to be involved in a remarkable variety of cellular processes and in pathological conditions including cancer and neurodegenerative diseases. This is documented by the evergrowing number of published results summarized in some recent reviews [2–4].

The specific enzymatic activity and wide binding capabilities of PDIA3 may allow its interaction with many other proteins. PDIA3 has been thought to be a participant in the mechanisms of cell protection against oxidative stress through its redox and chaperone activities but target substrates remain to be ascertained. Many studies suggest that PDIA3 can prevent oxidation of thiol residues and aggregation of target proteins which might result in a loss of functional and/or structural properties [5–7]. Expression of PDIA3 is increased in more than 70% of cancers and its expression has been associated with cell invasiveness, metastasis, and low overall survival. PDIA3 may play a role in the oncogenic transformation as a result of its ability to control intracellular and extracellular redox state via thiol-dependent reductase activity [8, 9]. Moreover, PDIA3 may be engaged in cellular signalling pathways involving reactive oxygen species: it may rescue the functional activity of target proteins that undergo redox modification and/or control the formation of macromolecular complexes involved in the adaptive response to oxidative stress.

In this contest, the identification of specific substances able to bind PDIA3 and to modify its properties will be very useful. Considering the high level of PDIA3 expression in several cancer types, specific inhibitors might offer new therapeutic tools, while the identification of ligands that can modulate and/or inhibit PDIA3 interaction with specific partners may be useful to selectively control cellular processes and signalling pathways involving PDIA3.

**Figure 1:**
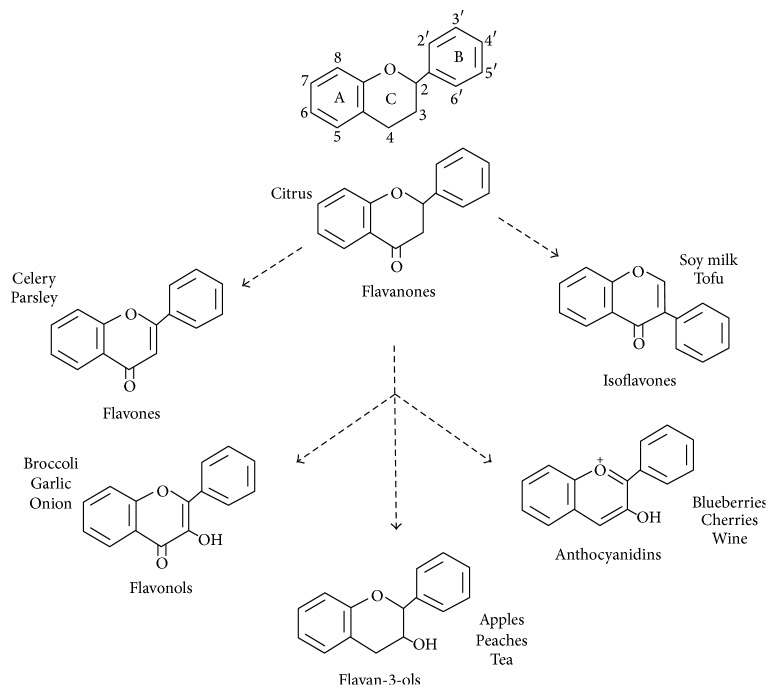
Flavonoid structures and occurrence. Arrows indicate biosynthetic path.

Flavonoids are a large class of polyphenolic compounds ubiquitous in plants and mostly present in fruits, vegetables, and plant-based beverages such as tea and wine [10]. Flavonoids are further subclassified in to flavones, flavonols, isoflavones, flavanones, flavanols, and anthocyanidins [11, 12] (Figure 1). These physiologically active compounds, which are produced via secondary metabolism, have well-known multiple health beneficial effects. Many studies have suggested an association between consumption of flavonoids-rich food or beverages and the prevention of many degenerative diseases, including cancer, neurodegeneration, and coronary heart disease and stroke [13–17]. Therefore, flavonoids represent one of the most attracting families of natural bioactive molecules and important components of a normal human diet.

The protection offered by flavonoids is believed to be due to their antioxidant activity. The aromatic rings of the flavonoid molecule allow the donation and acceptance of electrons from free radical species [18]. In addition, many polyphenols regenerate the traditional antioxidant vitamins, vitamin C and vitamin E [19], and act as metal chelators [20]. This latter property contributes to their antioxidant effects through the inhibition of free radical formation catalysed by transition metals [21]. However, the concentrations at which they can exert such antioxidant activity are unlikely achieved* in vivo*, as many of them have limited bioavailability and are extensively metabolized [22, 23].

It has been suggested that, in lower amounts, flavonoids as well as polyphenols may exert pharmacological activity within the cells, having the potential to modulate intracellular signalling pathways. Many polyphenols can induce antioxidant enzymes such as glutathione peroxidase, catalase, and superoxide dismutase and inhibit the expression of enzymes such as xanthine oxidase, which is involved in the generation of free radicals [24, 25]. However, for many of them the molecular and cellular bases of these activities are not well known. They can act by different targets including the regulation of cell signalling and the cell cycle, free radical scavenging, inhibition of angiogenesis, initiation of DNA repair mechanisms, apoptotic induction, and inhibition of metastasis. By affecting such pathways flavonoids have the ability to control cell proliferation and survival and to exhibit antiproliferative and antimutagenic properties and a remarkable anti-inflammatory activity [26–29].

Given the involvement of PDIA3 in the cellular response to stress as well as in cancer and neurodegeneration, we felt it is useful to undertake a screening study for assessing the interaction and impact on PDIA3 protein activity of several types of flavonoids. This study, which initially started analyzing the major catechins present in the extracts of green tea [30], has been expanded to various classes of flavonoids to verify if their activity was in some way connected to the modulation of PDIA3 functions.

The binding of several flavonoids to PDIA3 and their effects on the protein were tested by protein fluorescence quenching and differential scanning fluorimetry. Protein redox and DNA binding activities were also analyzed in the presence of flavonoids, with the aim to find specific molecules useful to control/modulate the cellular processes and signalling pathways involving the PDIA3 protein and to check for any relationship between the polyphenolic structure and the interaction with the protein.

## 2. Material and Methods

### 2.1. Chemicals

PBS solution, acrylamide, N-ethylmaleimide, DTT, GSSG, eosin isothiocyanate, and 19 different flavonoids (quercetin, 3-*O*-methyl quercetin, isoquercetin, quercitrin, rutin, morin, rhamnetin, isorhamnetin, fisetin, apigenin, apigenin-7-glucoside, luteolin-7-glucoside, kaempferol, eupatorin, eupatorin-5-methyl-ether, genistein, naringenin, cyanidin, and 6,2′,4′-trimethoxyflavone) were purchased from Sigma-Aldrich, EDTA (0.5 M solution pH 8.0) from IBI Scientific. SYPRO Orange was from Invitrogen.

### 2.2. Protein Expression and Purification

Human recombinant PDIA3 was cloned and expressed in* E. coli* strain BL21 using the expression vector pET21 (Novagen) as previously described [33]. The coding sequence for the second redox-active domain (*a*′ domain, residues 377–505) was amplified by PCR as previously described and cloned in the expression vector pET29 (Novagen) [34]. Recombinant proteins were expressed in* E. coli* strain BL21 and purified by ammonium sulphate fractionation, ion exchange, and heparin chromatography [34, 35]. Protein purification was evaluated by SDS-PAGE and concentration was determined spectrophotometrically (PDIA3 *ℇ*_280_ reduced form = 44,810 M^−1^ cm^−1^; *a*′ domain *ℇ*_280_ reduced form = 14,400 M^−1^ cm^−1^).

### 2.3. Fluorescence Quenching Measurements

Quantitative analysis of the interaction between individual flavonoids and PDIA3 was performed by fluorimetric titration. Fluorescence spectra were recorded using a SPEX-FluoroMax spectrofluorimeter (Horiba Scientific) from 300 to 500 nm with excitation at 290 nm using a 10 mm path length quartz fluorescence cuvette and under continuous stirring. The excitation and emission slits were both set to 5 nm and scan speed was 120 nm·min^−1^. Briefly, solution of PDIA3 (0.5 *μ*M) or *a* and *a*′ domains (1 *μ*M) in phosphate buffered saline (PBS) containing DTT 0.1 mM and EDTA 0.2 mM were titrated in quartz cuvette by stepwise additions, at 5 min time intervals, of individual flavonoid solution (1 mM in PBS/ethanol 50 : 50 v/v freshly prepared from a 20 mM stock solution in DMSO). Most of tested flavonoids can absorb light at the excitation and emission wavelengths. To minimize the inner-filter effect we limited the highest concentration reached in the titration test up to 10 *μ*M. All experiments were carried out at 25°C. Each spectrum was the mean of three repeated scans. Fluorescence spectra of appropriate blanks (flavonoids without proteins) were recorded under the same experimental conditions and subtracted from the corresponding flavonoid-protein system to correct the fluorescence background. Fluorescence intensities recorded at 338 nm were used for quenching analysis and data obtained for each flavonoid are the average of at least three independent titration experiments.

### 2.4. Differential Scanning Fluorimetry

Protein stability was tested by the fluorescence-based thermal stability assay or differential scanning fluorimetry (DSF) developed by Pantoliano and coworkers [36]. DSF records the fluorescence emission signal from the binding of a dye (SYPRO orange) to the exposed hydrophobic patches upon protein unfolding. Fluorescence measurements were performed using a CFX96 Connect RT-PCR instrument (BioRad). 18 *μ*l aliquots of protein solution (1 *μ*M), freshly diluted in Tris Buffered saline (50 mM TrisHCl, pH 8.0, 150 mM NaCl) containing 0.1 mM DTT, 0.2 mM EDTA, and 1 : 1000 dilution of SYPRO Orange (50 mM stock solution in DMSO), were distributed in a 96-well plate. Sample wells were addition of increasing concentration (up to 50 *μ*M) of individual flavonoid by adding 2 *μ*l of 10-fold concentrated stock solutions freshly prepared. As control, the protein solution was added with 2 *μ*l of the dilution buffer. A melting point analysis was recorded increasing the temperature from 20 to 95°C with an increment of 0.5°C and reading, at each temperature, the fluorescence in fluorescence resonance energy transfer mode (FRET). Each measure was the average of three sample wells reading and each DSF experiment was repeated at least three times. To calculate *T*_*m*_ values, DSF data from the melting curve were exported and fitted to Boltzman equation using GraphPad software [37]. Effect of flavonoids was evaluated comparing the *T*_*m*_ values in presence and absence of each molecule.

### 2.5. Determination of Protein Disulfide Reductase Activity

Disulfide reductase activity of ERp57 was monitored by sensitive fluorescent assay using dieosin glutathione disulfide (DiE-GSSG) as fluorogenic probe [38]. DiE-GSSG was synthesized by the reaction of eosin isothiocyanate with oxidized glutathione (GSSG) according to the method of Raturi and Mutus [38] with some modifications [30]. DiE-GSSG concentration was determined spectrophotometrically (*ℇ*_525_ = 88,000 M^-1 ^cm^−1^). Disulfide reductase activity was assayed in a reaction buffer containing 2 mM EDTA, 150 nM DiE-GSSG, and 5 *μ*M DTT in PBS. A 20 *μ*l aliquot of a stock solution of PDIA3 (1 *μ*M) was added to the reaction mixture and DiE-GSSG reduction was monitored at 545 nm with excitation at 520 nm, at 25°C with continuous stirring. Reductase activity was calculated as the initial velocity in fluorescence increase. To test the effect of flavonoids on PDIA3 activity, the protein stock solution was previously incubated for 15 minutes at room temperature with different concentrations of each flavonoid and then a 20 *μ*l aliquot subjected to assay in a reaction buffer containing the same concentration of the flavonoid.

### 2.6. Determination of DNA Binding Activity by EMSA

EMSA assay was performed using a 79-bp rich DNA fragment obtained by PCR as previously described [39]. Amplified DNA was end-labeled by using 5-fluorescein-modified primers. 10 ng of DNA fragment was incubated with 1 *μ*g of PDIA3 or *a*′ domain, in presence of increasing concentration of flavonoids (from 0 to 50 *μ*M). Controls were performed using DNA alone and in the presence of each flavonoid. All tests were performed in 20 *μ*l mixtures containing 20 mM Tris-HCl, pH 7.5, 50 mM NaCl, and 10% (v/v) glycerol. Samples were incubated for 1 hour at 25°C and then resolved on 5% polyacrylamide gels in 0,25% TBE buffer (22.5 mM Tris-borate, 5 mM EDTA, pH 8.3) at 150 V. Gel images were recorded using a ChemiDoc MP Imaging System (BioRad) equipped with an epi-blue excitation lamp and a 520 nm emission filter.

### 2.7. Statistical Analysis

Fluorescence quenching constant (*K*_SV_) values were given as means ± standard deviation and values of disulfide reductase activity were expressed in % of control sample ± relative standard deviation. All measurements were performed at least three times. Dunnett's test was used to compare the obtained reductase activity data with the activity of the untreated protein and a *p* value of <0.01 was considered as statistically significant.

### 2.8. Other Procedures

Proteins were analyzed by SDS-PAGE. Samples were added with 10 mM N-ethylmaleimide before dilution with concentrated SDS-sample buffer to prevent any reactivity of free thiol groups.

## 3. Results and Discussion

### 3.1. Study of the Flavonoids-PDIA3 Interaction by Fluorescence Analysis

**Figure 2:**
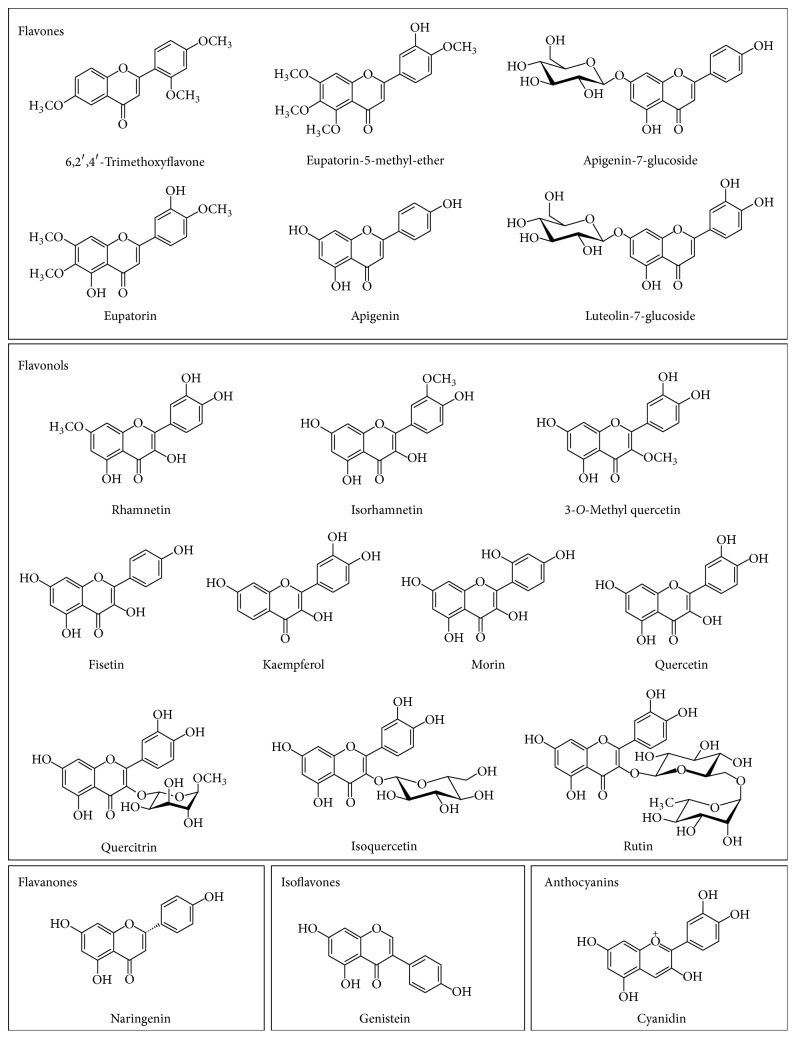
Molecular structure of tested flavonoids.

We started a screening analysis to find molecules which specifically bind PDIA3 and can inhibit its redox activity and/or the binding to other proteins or DNA sequences [30]. Such substances could be useful to modulate the biological functions of PDIA3. In this study the interaction of different flavonoids with PDIA3 and their effects on protein reductase activity were evaluated. A number of flavonoids, comprehending flavones, flavonols, and several derivatives, which differ in terms of skeleton structure as well as hydroxyl, methoxyl, and other substituted groups, were analyzed (Figure 2). The interaction was investigated by quenching analysis of PDIA3 intrinsic fluorescence mainly due to the presence of three tryptophan residues. They differ from each other in solvent or quencher accessibility and can unequally contribute to the protein fluorescence. One tryptophan residue (W279) is buried in a hydrophobic pocket in the *b*′ domain whereas the other two (W56 and W405) are present on the protein surface close to the thioredoxin-like active sites within *a* and *a*′ domains, respectively.

Quenching analysis was performed on PDIA3 (0.5 *μ*M), in the reduced form, by adding stepwise increasing concentration of each molecule and recording the protein fluorescence spectra. For some flavonoids the analysis was extended to isolate *a*′ domain (1 *μ*M), always in the reduced form. Quenching effect on protein and Stern-Volmer quenching constant (*K*_SV_) were calculated from the fluorescence intensities at 338 nm of protein alone (*F*_0_) and in the presence of increasing concentration of each ligand molecule (*F*) using the Stern-Volmer equation [40]:

(1) $$\begin{array}{c} \frac{F_{0}}{F}=1+K_{SV}\left[\;L\;\right], \end{array}$$

where *L* is the ligand concentration. For each ligand molecule the Stern-Volmer quenching constant was obtained by linear regression of plots of *F*_0_/*F* versus [*L*].

**Figure 3:**
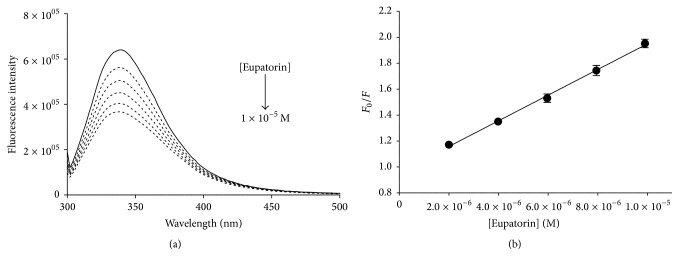
(a) Fluorescence quenching spectra of reduced PDIA3 alone (solid line) and after stepwise addition of eupatorin (dotted line) (pH 7.4, 25°C, and *λ*_ex_ = 290 nm). [PDIA3] = 0.5 × 10^−6^ M, [eupatorin] = 2 × 10^−5^ M final concentration. (b) Stern-Volmer plot of quenching data as mean of at least three independent experiments (standard deviations were better than 10% and correlation coefficient was better than 0.99).

Representative fluorescence spectra of PDIA3 in the presence of increasing concentration of eupatorin are showed in Figure 3(a). A different degree of quenching was observed in presence of tested molecules. The decrease of fluorescence may indicate that the microenvironments of tryptophan residues in PDIA3 were altered due to the interaction with tested compounds. No evident spectral shift was noticed in fluorescence spectra of PDIA3 after the additions of all tested flavonoids, suggesting that these substances do not induce any evident change in protein conformation.

**Figure 4:**
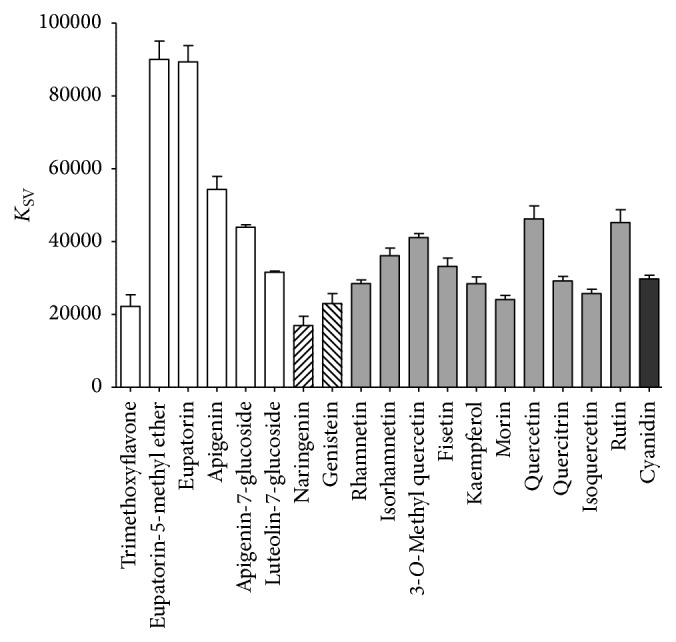
Estimated *K*_SV_ values of all tested flavonoids obtained by fluorescence quenching analysis of reduced PDIA3 [0.5 × 10^−6^ M] in presence of increasing concentration of flavonoids (up to 2 × 10^−5^ M). Data are reported as mean and standard deviation of at least three independent experiments.

Fluorescence quenching data were analyzed using the Stern-Volmer equation. The Stern-Volmer plot for PDIA3, in presence of eupatorin, is displayed in Figure 3(b) while the estimated *K*_SV_ values of all tested flavonoids are summarized in Table 1 and graphed in Figure 4.

The Stern-Volmer quenching constant may be expressed as *K*_SV_ = *K*_*q*_ *τ*_0_, where *K*_*q*_ is the quencher rate coefficient and *τ*_0_ is the average lifetime of the emissive excited state of the protein in the absence of the quencher. Based on a typical value of *τ*_0_ of the order of 10^−8^ s [40, 41], the values of *K*_*q*_ can be estimated and are reported in Table 1. The values of *K*_*q*_ observed for flavonoids-PDIA3 interaction are much greater than 2.0 × 10^10^ M^−1^ s^−1^, the maximum diffusion collision quenching rate constant reported for the interaction of various kinds of quencher with proteins [42], suggesting that the quenching is not the result of a dynamic collision but it occurs via formation of complexes between the protein and flavonoids. This interaction should involve regions of the protein near the two tryptophan residues, W56 and W405, more exposed to the solvent and close to the redox-active sites.

In static quenching, various equations expressing the relationship between fluorescence intensity and quencher concentration have been described for the evaluation of the binding constant and the number of binding sites. However, the analysis of protein-ligand binding, using fluorescence quenching, may be affected by a number of pitfalls that cannot be easily overcome.

The apparent binding constants (*K*_*b*_) and the number of binding sites (*n*) were calculated using the equation described by Bi et al. [31], as previously reported [30]:

(2) log⁡F0−FF=nlog⁡Kb−nlog⁡1Lt−nF0−FPt/F0,

where *F*_0_ and *F* are the fluorescence intensities at 338 nm before and after the addition of the quencher and [*L*] and [*P*_*t*_] are the ligand and the total protein concentrations, respectively. The number of binding sites (*n*) and the binding constant (*K*_*b*_) were obtained by plotting log⁡((*F*_0_ − *F*)/*F*) versus log⁡(1/([*L*_*t*_] − *n*(*F*_0_ − *F*)[*P*_*t*_]/*F*_0_)) using the reiterative calculation process described by Sun et al. [32], assuming a similar affinity for each binding site. The dissociation constant was calculated from the binding constant (*K*_*d*_ = 1/*K*_*b*_).

The estimated *K*_SV_, *K*_*b*_, and *K*_*d*_ values that characterize the interaction of PDIA3 with tested flavonoids are summarized in Table 1. Although the protein can bind most of the tested substances, with an estimated dissociation constant within the 10^−5^ M range, some of them showed a better affinity. Molecules characterized by the highest binding constants are eupatorin and eupatorin-5-methyl ether, with a *K*_*d*_ around 1.0 × 10^−5^ M, followed by apigenin, with a *K*_*d*_ of 1.7 × 10^−5^ M.

The use of several flavonoids differing in chemical structure and functional groups allows us to make some considerations about the ligand structures and their affinity for PDIA3. Comparing the structure of the molecules with higher affinity, they share the same basic flavone backbone: eupatorin-5-methyl ether and eupatorin are characterized by the presence of both hydroxyl- and methoxyl- groups, while the flavone apigenin that has only hydroxyl groups, two on the A ring and one on the B ring, has a lower affinity. 6,2′,4′-Trimethoxyflavone, a flavone with only methoxyl groups, showed the lowest affinity for the protein. In this respect, the specific degree of polarity of the flavone backbone seems to play an important role for the interaction with PDIA3. The addition of a sugar moiety, like glucose in apigenin-7-glucoside, negatively affects the binding compared to apigenin. The affinity is even lower analyzing luteolin-7-glucoside, which has one more hydroxyl group on the B ring. These observations suggest that a specific degree of hydrophilicity is required to stabilize the interaction of the flavone backbone with the protein.

**Figure 5:**
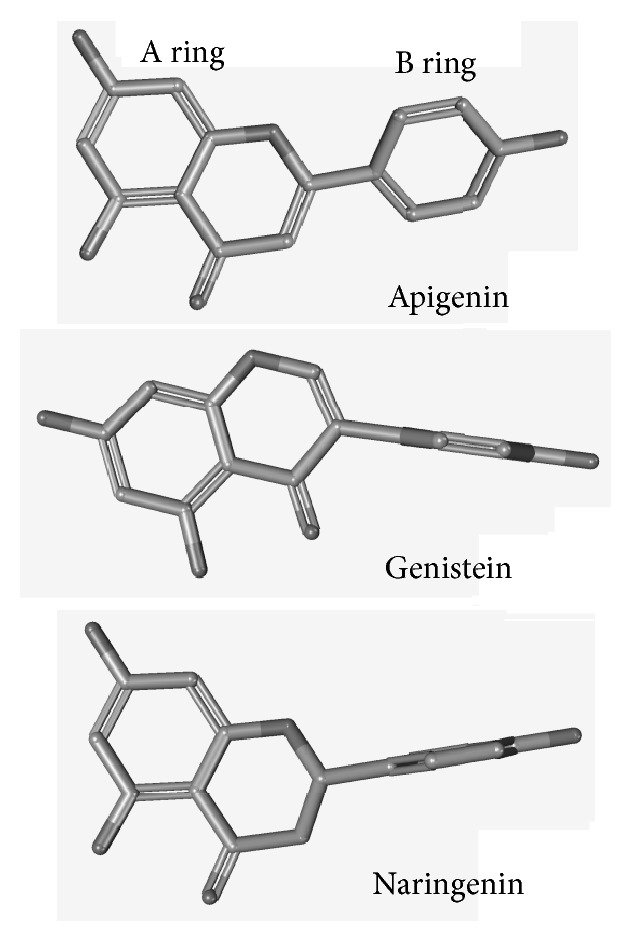
3D models of default conformers for apigenin (CID 5280443), genistein (CID 5280961), and naringenin (CID 932) generated by PubChem (https://pubchem.ncbi.nlm.nih.gov/).

Apigenin, genistein, and naringenin share similar functional groups but differ for the position and orientation of the B ring. In naringenin the B ring on the C2 position is differently oriented with respect to A and C rings while it is also differently positioned (C3) in genistein (3D models of default conformers are shown in Figure 5). The flavanon naringenin showed a much lower binding affinity compared to apigenin; hence, it can be hypothesized that the more parallel orientation between the aromatic rings A and B, mainly evident in flavones, is an important feature to the binding to PDIA3. The isoflavone genistein also showed a lower affinity compared to apigenin, stressing out the importance of a specific position and orientation of the B ring for the interaction with PDIA3.

The analysis of different flavonols showed dissociation constants between 2.0 × 10^−5^ M and 4.0 × 10^−5^ M. Flavonols, different from flavones, are characterized by a B ring that, in the most stable conformation, is almost perpendicular to the A ring due to the presence of a hydroxyl group linked to the C3 position; this may explain their lower affinity. As above reported for flavons, we can observe some differences in the affinity within the analyzed flavonol. Among flavonols sharing only hydroxyl groups as substituents, quercetin and fisetin showed a slight better affinity compared to kaempferol and morin. These flavonols differ in the specific position of hydroxyl groups, and it could be hypothesized that a hydroxyl group sited at 5 positions on A ring and unsubstituted C2^'^ hydroxyl group on B ring have a positive effect on the dissociation constant. Flavonols with a similar structure but characterized by the presence of a methoxyl group in three different positions (rhamnetin, isorhamnetin, and 3-*O*-methyl quercetin, with the –OCH_3_ group sited on the A, B, and C rings, resp.) differently bind PDIA3, with 3-*O*-methyl quercetin showing a higher affinity with respect to rhamnetin and isorhamnetin. Therefore, beside the orientation of aromatic rings, the specific distribution of functional groups on the flavonol backbone structure plays an important role to stabilize the interaction with the protein.

Finally, the effect on the flavonoid-PDIA3 binding of a sugar moiety, linked to the backbone structure, was tested. As above reported, the presence of a glucose moiety on the C7 position has a negative effect on the binding of flavones apigenin-7-glucoside and luteolin-7-glucoside compared to the unglycosylated form. For flavonols we observed a similar behavior. In fact, the addition of a single carbohydrate linked to the C3 position (isoquercetin and quercitrin) lowers the binding affinity for the protein compared to quercetin, the corresponding unglycosylated flavonoid. Thus, the presence of a carbohydrate moiety has a negative effect on the binding to the PDIA3 protein, probably because this increases the polarity of one side of the flavonoid molecule. In fact, as above emphasized, the flavonoids showing the best affinity are eupatorin and eupatorin-5-methyl ether, both characterized by a specific degree of polarity, with few hydroxyl groups and a larger number of methoxyl groups, uniformly distributed around the backbone structure. However, increasing the number of carbohydrate linked to the C3 position raises the affinity as results in the similar binding properties of rutin and quercetin. This positive effect may be the result of an increased size of the molecule and the number of interactions that can be established with the protein.

For some flavonoids, the binding analysis was extended to the isolated *a*′ domain. Similar values for the binding constants were estimated, with some flavonoids showing a slightly better affinity for the whole protein respect to the isolated domain. Even in this case, the molecules that show the greatest affinity are the flavones eupatorin, eupatorin-5-methyl ether, and apigenin (data not shown). PDIA3 and the homologous isoform PDIA1, although similar in overall domain structure and active-site motifs, carry out different functions that are the result of specialized substrate binding properties. These differences are mainly related to the C-terminal domains *b*′ and *a*′ that show the lowest homology between the two isoforms [43, 44]. Our findings indicate that flavonoids can effectively bind the PDIA3 isoform through its C-terminal *a*′ domain, as we previously reported for galloylated catechins [30].

Fluorescence quenching data suggest that flavonoids' interaction with the protein involves mainly the tryptophan residues near to the redox-active sites. Although PDIA3 contains two redox-active domains, both potentially able to interact, data analysis estimates a single binding site for each molecule. It could be possible that differences in the affinity of the two binding sites introduce a further pitfall in math calculation of binding stoichiometry. However, as previously hypothesized for catechins-PDIA3 interaction, we suggest that the binding of the first molecule to one of the redox domain prevents the binding of a second molecule to the other redox domain, as a result of steric hindrance and/or changes in the local environment that hinder the second site [30].

### 3.2. Effect of Flavonoids on the PDIA3 Stability

**Figure 6:**
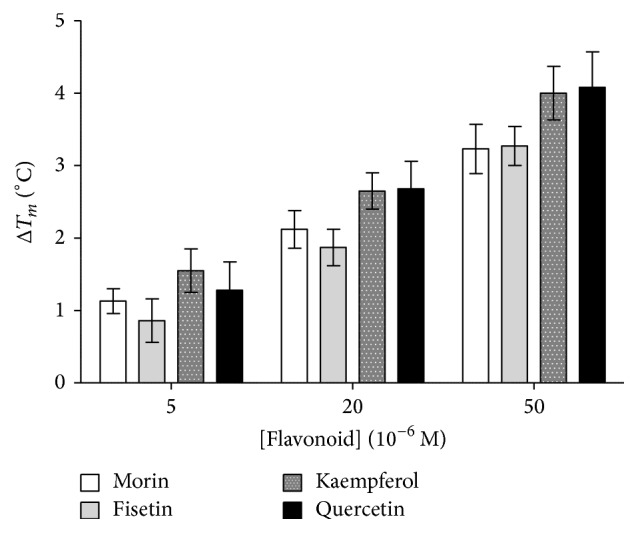
Increase in melting temperature (*T*_*m*_) of PDIA3 calculated by differential scanning fluorimetry of reduced PDIA3 [1 × 10^−6^ M] in presence of indicated concentrations of selected flavonoids. Plots are displayed as mean and standard deviations of at least three independent measurements.

The effect of flavonoids on the protein stability was evaluated by DSF. Most of the tested molecules showed no or limited effect, but quercetin, morin, kaempferol, and fisetin can stabilize the protein in a concentration-dependent manner. The *T*_*m*_ value calculated for PDIA3 by scanning fluorimetry was increased more than three degrees at 50 *μ*M concentration of these flavonoids (Figure 6). Apigenin, isorhamnetin, rhamnetin, genistein, and cyanidin produced a similar, but less pronounced, effect and mainly at the highest concentration. Quercetin, morin, kaempferol, and fisetin, even if characterized by a lower affinity compared to the flavones eupatorin and eupatorin-5-methyl ether, share a protective effect against thermal denaturation. This may be the result of their specific structure, rich in hydroxyl groups distributed around the flavonol backbone; in fact, the stabilizing effect of polyols on protein structure has been widely described [45, 46]. Glycosylated flavonoids do not seem to have such stabilizing properties, probably because of their different size and solubility that may change the interaction with protein and solvent.

### 3.3. Effect of Flavonoids on the PDIA3 Redox Activity

**Figure 7:**
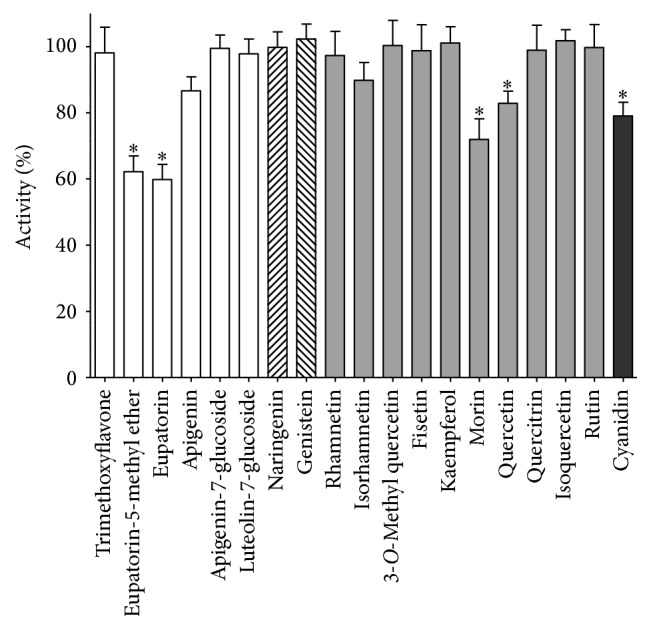
Comparison of flavonoid effect (at 20 *μ*M concentration) on PDIA3 reductase activity. Plots are displayed as mean and standard deviations of at least six independent measurements. Data were analyzed by Dunnett's test comparing PDIA3 activity in presence of each flavonoid with the activity of untreated protein. Significant differences (*p* < 0.01) are marked with asterisks.

To verify if the interaction between flavonoids and PDIA3 may have an effect on protein functions, the disulfide reductase activity was tested. For most of the molecules analyzed the effect on the redox activity of the protein was negligible. However, some flavonoids, in particular the flavones eupatorin and eupatorin-5-methyl ether, showed an evident inhibitory effect, up to 40%. Others, such as morin, quercetin, and cyanidin, had a less marked inhibition, approximately 20% (Figure 7). Apigenin, which showed a binding affinity close to eupatorin, had no evident effect on the protein activity. It is possible that the less polar degree that characterizes eupatorin and eupatorin-5-methyl ether may play a significant role. It should be also noted that more hydrophilic molecules like morin, quercetin, and cyanidin share a certain inhibitory effect indicating a more complex pattern of interaction with the protein. On the basis of these results, we hypothesize that, at least for those molecules showing a marked inhibitory effect on redox activity, the contact region of interaction between the flavonoid structure and PDIA3 should involve the active site. However, since we used a simple substrate like DiE-GSSG to measure the redox activity, we cannot exclude that the inhibitory effect of all flavonoids could be different towards more physiological substrates.

### 3.4. Effect of Flavonoids on the DNA Binding Property of PDIA3

We also evaluated by EMSA analysis the effect of all analyzed flavonoids on the DNA binding property of the protein. None of tested flavonoids showed an appreciable effect on the DNA binding activity of the protein, different from what previously reported for galloylated catechins [30]. In that case, we hypothesized that the galloyl moiety on the C3 position could prevent the DNA binding activity interacting with a *β*-strand region close to the redox-active site. Upon protein oxidation, such region can undergo to a conformational change that is essential for the interaction with the DNA [34]. In this report none of the molecules analyzed have such effect. As proposed above, we suggest that flavonoids interact with the same protein region containing the tryptophan residues close to the active sites. However, this interaction may differently extend depending on the flavonoid structure and on specific contacts between flavonoids functional groups and amino acidic residues on the protein surface.

## 4. Conclusions

**Figure 8:**
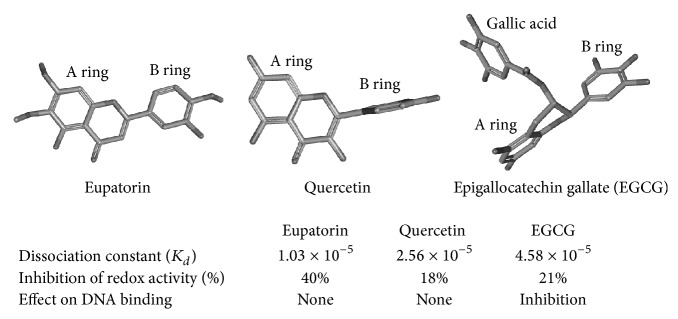
3D models of default conformers for eupatorin (CID 97214), quercetin (CID 5280343), and epigallocatechin gallate (CID 65064) generated by PubChem. The estimated *K*_*d*_ values that characterize the interaction of PDIA3 with the three flavonoids, the percentage of inhibition of protein redox activity, and the effect on DNA binding are also reported (epigallocatechin gallate data were from Trnková et al. [30]).

The interaction of different flavonoids with PDIA3 and their effect on protein reductase activity were evaluated in a comparative analysis of protein intrinsic fluorescence quenching. Eupatorin and eupatorin-5-methyl ether, flavones both characterized by a mild polarity for the presence of several methoxyl groups, showed the highest affinity for PDIA3 with a *K*_*d*_ near to 1.0 × 10^−5^ M. The same molecules showed a noticeable inhibitory effect on disulphide reductase activity of PDIA3 but did not significantly affect its DNA binding activity. The flavone backbone structure is characterized by a more stable conformation where B and A rings are almost parallel (Figure 8). This structure, associated with a definite degree of polarity, due to the presence of several methoxyl groups, seems to be an important feature to determine a good affinity towards PDIA3. On the other hands, flavonols, which are characterized by a B ring mainly positioned perpendicular to the A ring, share a lower affinity. Therefore, the different orientation of the B ring can modify the interaction with the protein. Our data suggest that, different from what observed for flavones, the binding of flavonols varies on the basis of the presence and specific position of functional groups linked to the backbone. Only morin and quercetin, among flavonols, showed some inhibitory effect on PDIA3 redox activity even if to a lesser extent than flavones eupatorin and eupatorin-5-methyl ether. None of tested molecules prevent the binding of PDIA3 to DNA.

We can hypothesize that flavones and flavonols interact with a similar region of the protein involving the tryptophan residues close to the redox site. Given that PDIA3 does not contain any evident deep cavity or slot where this kind of ligands can bind, the binding of flavonoids may occur mainly via a flat interaction with the protein surface. Therefore, the planarity of the molecule as well as the number and specific position of its functional groups (hydroxyl, methoxyl, and carbohydrates) will definitively play a major role to determine the affinity for the protein. This binding site is probably similar but not equivalent to that of green tea galloylated catechins, which, as previously reported, can bind to PDIA3 and to its isolated *a*′ domain and prevent the interaction with DNA [30]. The presence in these molecules of a galloyl moiety linked to the C3 position is probably responsible for a different type of interaction that may extend to a protein region important to the contact with DNA (Figure 8). Another polyphenolic compound, the flavonolignan silibinin, has been previously showed to bind PDIA3 with a dissociation constant within micromolar range [47]. Silibinin higher affinity may be explained by its larger size that allows a better interaction with the surface of the protein. Since silibinin has no effect on the redox activity and DNA binding capability of PDIA3, we can expect that its binding involves a further different region close to the active site.

In conclusion, eupatorin and eupatorin-5-methyl ether represent leading compounds for the binding to PDIA3 and for the inhibition of its redox activity. Further experiments are required to better characterize the effect of flavonoids on PDIA3 and to understand if some of the biological activities of these compounds are depending on the interaction with PDIA3. Previously studies showed that eupatorin exhibits antiproliferative activity and apoptosis induction preferentially in cancer cells [48, 49]. In addition, eupatorin behaves as nonspecific inhibitor of several protein kinases, but its anticancer effect can also depend on the ability to interfere with angiogenesis through inhibition of VEGFRs [50]. A recent study provides evidence regarding antiproliferative action of eupatorin-5-methyl ether through a pathway that involves activation of CYP1 enzymes [51]. Although previous reports demonstrated antitumor properties of eupatorin-5-methyl ether, the mechanism of action remains poorly understood [52]. Since these flavones and PDIA3 are both involved in proliferative and carcinogenic processes, our* in vitro* findings on their interaction suggest that some of the biological effects of flavones can be mediated by modulation of PDIA3 activity. Additionally, this study will help to define and identify compounds to be used as selective inhibitors/modulators of PDIA3 biological activities.

## Figures and Tables

**Table 1:** Stern-Volmer constant (*K*_SV_), bimolecular quenching rate constant (*K*_*q*_), number of binding sites (*n*), binding constant (*K*_*b*_), and dissociation constant (*K*_*d*_) of several flavonoid-PDIA3 systems.

Flavonoid	*K*_SV_ (M^−1^)	*K*_*q*_ (M^−1^ s^−1^)	*n*	*K*_*b*_ (M^−1^)	*K*_*d*_ (M)
Flavone					
Trimethoxyflavone	22209 ± 484	2.22 · 10^12^	0.90	1,81 · 10^4^	5.52 · 10^−5^
Eupatorin-5-methyl ether	90016 ± 1875	9.00 · 10^12^	1.08	9.04 · 10^4^	1.11 · 10^−5^
Eupatorin	89362 ± 688	8.94 · 10^12^	1.16	9.67 · 10^4^	1.03 · 10^−5^
Apigenin	54332 ± 1185	5.43 · 10^12^	1.08	5.87 · 10^4^	1.70 · 10^−5^
Apigenin-7-glucoside	43961 ± 673	4.40 · 10^12^	1.06	4.73 · 10^4^	2.11 · 10^−5^
Luteolin-7-glucoside	31601 ± 484	3.16 · 10^12^	1.06	3.46 · 10^4^	2.89 · 10^−5^
Flavanone					
Naringenin	16900 ± 352	1.69 · 10^12^	1.05	1.73 · 10^4^	5.78 · 10^−5^
Isoflavone					
Genistein	22953 ± 435	2.30 · 10^12^	1.09	2.44 · 10^4^	4.10 · 10^−5^
Flavonol					
Rhamnetin	28462 ± 295	2.85 · 10^12^	1.03	3.01 · 10^4^	3.32 · 10^−5^
Isorhamnetin	36109 ± 1271	3.61 · 10^12^	0.96	3.25 · 10^4^	3.08 · 10^−5^
3-O-Methyl-quercetin	41110 ± 794	4.11 · 10^12^	1.09	4.57 · 10^4^	2.19 · 10^−5^
Fisetin	33164 ± 542	3.32 · 10^12^	1.08	3.67 · 10^4^	2.71 · 10^−5^
Kaempferol	28423 ± 982	2.84 · 10^12^	0.97	2.60 · 10^4^	3.85 · 10^−5^
Morin	24063 ± 265	2.41 · 10^12^	1.04	2.56 · 10^4^	3.90 · 10^−5^
Quercetin	46226 ± 1406	4.62 · 10^12^	0.88	3.90 · 10^4^	2.56 · 10^−5^
Quercitrin	29211 ± 483	2.92 · 10^12^	1.07	3.23 · 10^4^	3.10 · 10^−5^
Isoquercetin	25738 ± 367	2.57 · 10^12^	1.08	2.91 · 10^4^	3.44 · 10^−5^
Rutin	45197 ± 465	4.52 · 10^12^	0.96	4.55 · 10^4^	2.20 · 10^−5^
Anthocyanin					
Cyanidin	29761 ± 635	2.98 · 10^12^	0.99	2.93 · 10^4^	3.42 · 10^−5^

Data were calculate from fluorescence quenching analysis using 0.5*∗*10^−6^ M PDIA3 in reduced conditions (pH 7.4, 25°C) and increasing concentration (0 to 10*∗*10^−6^ M) of different flavonoids. *K*_SV_ are reported as mean and standard deviation of at least three independent experiments. The number of binding sites (*n*), the binding constant (*K*_*b*_), and the dissociation constant (*K*_*d*_) were estimated using the equation described by Bi et al. [31] and the reiterative calculation process described by Sun et al. [32].
